# A low follicle-stimulating hormone level is a protective factor for non-alcoholic fatty liver disease in older men aged over 80

**DOI:** 10.1186/s12877-021-02490-6

**Published:** 2021-10-12

**Authors:** Yunxia Zhu, Jun Xu, Xiaoyan Zhang, Yingying Ke, Guoxiang Fu, Qihao Guo

**Affiliations:** 1 Department of Geriatrics, Shanghai Jiao Tong University Affiliated Sixth People’s Hospital, Shanghai, 200233 China; 2 Department of Geriatrics, the Tenth People’s Hospital of Shanghai, Tongji University, Shanghai, China

**Keywords:** Follicle-stimulating hormone, Non-alcoholic fatty liver disease, Aged men

## Abstract

**Purpose:**

Recent studies have suggested the significant relationship between follicle-stimulating hormone (FSH) and non-alcoholic fatty liver disease (NAFLD) in postmenopausal women. However, it is unknown whether FSH impacts the risk of NAFLD in men. This study aimed to investigate the association between serum FSH levels and NAFLD in elderly Chinese men aged 80–98, a particular group with worse outcomes of NAFLD.

**Patients and methods:**

A cross-sectional analysis was performed in 444 subjects in a geriatric health center. The highest quartile of serum FSH was used as reference. Hepatic steatosis was defined according to the results of liver ultrasound. Fibrosis-4 (FIB-4) Index **>** 2.67 was defined as advanced fibrosis.

**Results:**

Based on liver ultrasound, 108 (24.3%) subjects had NAFLD. FSH level were negatively correlated with total testosterone, estradiol, nutritional risk, and the prevalence of high education level (all *P* < 0.01), and positively correlated with age, luteinizing hormone, alanine aminotransferase and aspartate aminotransferase (all *P* < 0.05). The correlation between FSH and body mass index or antihypertensive drug usage was marginally significant (*P* = 0.057; *P* = 0.066, respectively). The percentage of subjects with NAFLD had a trend to increase following the quartiles of serum FSH (20.0% in quartile 1, 18.2% in quartile 2, 27.3% in quartile 3, and 31.6% in quartile 4). After adjustment for common pathogenic risk factors, nutritional risk, and other sex hormones, serum FSH were progressively associated with odds ratios for NAFLD. The adjusted odds ratios and 95% CIs for quartile 1, quartile 2, and quartile 3, compared with quartile 4 were 0.132 (0.034–0.516), 0.190 (0.052–0.702), and 0.404 (0.139–1.173), respectively. Obesity was not involved in the potential negative role of circulating FSH on the risk of NAFLD in our population. Furthermore, our results revealed no significant association between FSH and advance fibrosis, the OR (95% CI) for advanced fibrosis was 1.018 (0.983–1.054) (*P* = 0.316) after adjusting for the potential covariates, although a positive correlation of FSH and FIB-4 score was observed (*r* = 0.325, *P* = 0.001).

**Conclusion:**

Low FSH level may decrease the risk of NAFLD in elderly Chinese men. These findings warrant replication in more extensive studies.

## Introduction

Non-alcoholic fatty liver disease (NAFLD) is defined as the presence of excessive fat deposition in the liver when the secondary etiologies are excluded. It is widely recognized as a major public health problem and is the most rapidly growing contributor to liver mortality and morbidity, according to the 2017 Global Burden of Disease study [1]. NAFLD is observed in 29.62% Asian population regardless of the diagnostic method [2]. It not only elevates liver enzyme levels and causes cirrhosis, but it also has a considerable impact on extrahepatic disorders, including diabetes, cardiovascular disease, metabolic syndrome, and other arteriosclerosis diseases [3, 4].

The inner mechanisms underlying NAFLD are far from being completely clarified. Many controversies persist in NAFLD research and clinical investigation although some progress has been made. Thus, finding the cure for this widespread illness is still a challenge [5]. A growing body of evidence suggests that sex steroid hormones might play a role predisposing individuals to NAFLD [6]. For example, lower testosterone levels were associated with an increased risk of NAFLD in men [7, 8], and estradiol is reported to be one of the protective factors for NAFLD in healthy men [9]. Recently, the roles of follicle-stimulating hormone (FSH) in metabolic diseases (obesity, dyslipidemia, metabolic syndrome, diabetes, and atherosclerotic cardiovascular disease), which are closely related to NAFLD, have been gradually revealed [10–13]. Consequently, the connection between FSH and NAFLD was also observed in postmenopausal women. Women with lower FSH levels had an increased risk of NAFLD based on liver ultrasonography [14]. Besides circulating FSH levels, the diurnal rhythm of FSH was reported to associate with NAFLD in a small sample size study (71 subjects) of an elderly population [15].

However, to our knowledge, all previous studies reporting the association of circulating FSH levels with NAFLD have been conducted in postmenopausal women or a mixed population [14, 15]. It remains unclear how FSH levels associated with NAFLD in men, especially older men. Older age and male sex have been associated with shorter survival and a higher incidence of hepatocellular carcinoma (the worst outcomes of NAFLD) [16]. Thus, it is of clinical relevance to investigate the association of FSH with NAFLD in older men. To address this question, we analyzed the association between serum FSH levels and NAFLD in Chinese male subjects ≥80 years and focused on several possible explanatory factors, including common and uncommon pathogenic risks.

## Material and methods

### Study design

**Fig. 1:**
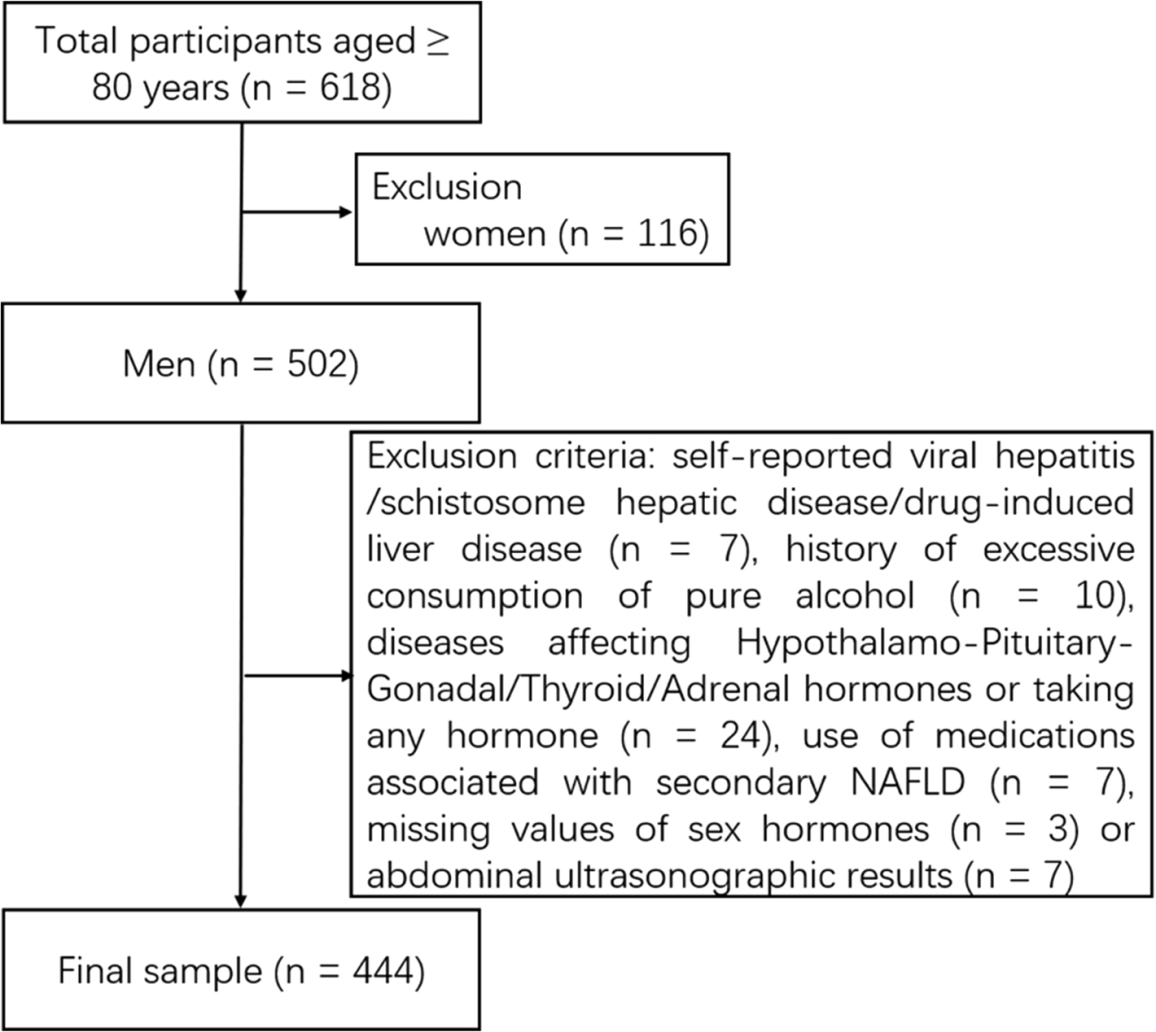
Flowchart for subjects enrolled in this study

This was a cross-sectional single-center study assessing FSH, metabolic risks, and aging conducted at the Shanghai Jiaotong University Affiliated Sixth People’s Hospital. The registration number of this study was ChiCTR1800018015 on www.chictr.org.cn. We advertised our study in the outpatient area of the Geriatrics Department and recruited old subjects who took part in health examination at the Department of Geriatrics, between January 2019 and December 2019. The majority of the participants were retired professors living in Shanghai, one of the most important metropolises in China. We recruited subjects based on the following criteria: (i) adults, ≥ 80 years of age; (ii) no communication problems; (iii) no acute illness and ending carcinomatous cachexia. A total of 618 subjects (aged 80–98 years) were included in this investigation. We excluded participants who were female (*n* = 116), with schistosome hepatic disease, self-reported viral hepatitis or drug-induced liver disease (*n* = 7), had a history of excessive consumption (> 20 g/day) of pure alcohol (*n* = 10), with diseases affecting the Hypothalamo-Pituitary-Gonadal/Thyroid/Adrenal hormones or taking any hormone (*n* = 24), who used medications associated with secondary hepatic steatosis (corticosteroids, tamoxifen, amiodarone, and methotrexate) (*n* = 7). Subjects with missing values of sex hormones (*n* = 3) or abdominal ultrasonographic results (*n* = 7) were also excluded. Finally, 444 male subjects were included in this cross-sectional analysis (Fig. 1).

### Measurements

All participants reported data on demographics, lifestyle variables (smoking status and exercise habits), history of personal disease and medication use (in the past seven 7 days), and anthropometric parameters were measured. We also assessed the nutritional status and functional health as the participants in this study were very old men, who often were at higher nutritional risk and disability. Education was dichotomized with a cutoff of college graduation versus no college graduation. Marital status was categorized based on living with a partner/married or other. Smoking history was classified into non-, ex-, and current smokers. There were only two current smokers in our population, and we pooled current and ex-smokers as current/ex-smokers in the result section. We designated exercise using two categories: routine exercise (moderate to strenuous intensity, three times a week or more frequently) and non-routine exercise. Nutritional status was assessed by the Short-form of Mini Nutritional Assessment (MNA-SF), a widely used nutritional screening tool [17]. In brief, six questions selected from the MNA were included. We assessed body mass index (BMI), recent weight loss, appetite or eating problems, acute illness/psychological stress, mobility impairment, and dementia or depression. Each question was rated from 0 to 2 or 3, with a total maximum score of 14. Subjects with scores ≤11 were considered at nutritional risk/malnutrition. Anthropometric data included height, weight, and waist circumference (WC). Height and weight were measured with the participants barefoot and in light clothing using the height and weight scale to the nearest 0.1 cm or 0.1 kg, respectively. BMI was calculated as the weight in kilograms divided by the height in meters squared. The WC was measured at the middle point between the rib cage and the iliac crests. All measurements were performed in duplicate, and the results were averaged for analysis. Blood pressure (BP) was measured after a 10 min rest in a sitting position using a standard mercury sphygmomanometer.

### Liver ultrasound

Subjects with NAFLD were characterized based on Guidelines for the diagnosis of NAFLD proposed by the Asia-Pacific Working Party [17]. Based on the established ultrasound criteria for fatty liver by abdominal ultrasonography, NAFLD was clinically defined. Radiologists were blinded to the clinical information of the subjects.

### Laboratory measurements

Fasting venous blood samples were collected from all subjects between 7:00 and 8:00 AM. The serum lipid profile was assayed by standard enzymatic methods using a Hitachi 747 analyzer (Castle Hill, NSW, Australia). Plasma glucose concentrations were measured using the glucose oxidase method. The hemoglobin A1c (HbA1c) value was measured by high-performance liquid chromatography (Bio-Rad Laboratories, Hercules, CA, USA). Fasting serum insulin (FINS) was detected using radioimmunoassay kits according to the manufacturer’s instructions (Beijing North Institute of Biological Technology, Beijing, China). Serum alanine aminotransferase (ALT) and aspartate aminotransferase (AST) were measured using the bromocresol green method (Shanghai Kehua Bio-Engineering Co, Ltd., China). The FIB-4 index was calculated using the formula: age (yrs) × AST[U/L] / (platelet[10^9^/L] × (ALT[U/L]^1/2^). For inflammatory biomarkers, the following techniques were used: immunoturbidimetry for high-sensitivity C-reactive protein (hsCRP), immunofluorescence for procalcitonin (PCT). Sex hormones levels, including total testosterone (TT), estradiol (E_2_), FSH, and luteinizing hormone (LH), were assayed by a chemiluminescence method (kits from Abbott GmbH & Co. KG, Wiesbaden, Germany) with inter- and intra-assay coefficients of variations of < 10%. The normal range was 6.68 to 25.7 nM for TT, 41.4 to 159 pM for E_2_, 1.5 to 12.4 IU/L for FSH, and 1.7 to 8.6 IU/L for LH.

### Statistical analysis

The data were tested for normality using the Shapiro-Wilk test, and continuous variables were presented as the mean ± standard deviation (SD) for normally distributed variables or the median (interquartile range) for skewed variables. For normally distributed and skewed variables, differences between the non-NAFLD and NAFLD groups were evaluated with the Student’s t-test or Mann–Whitney U-test, and the differences among FSH quartiles were analyzed by one-way analysis of the variance (ANOVA) or the Kruskal-Wallis test, respectively. Categorical variables were expressed as percentages (%), and intergroup comparisons were analyzed using the chi-square (χ^2^) test. Pearson or spearman analysis was used to assess the relationship between FSH levels and various parameters. The association between FSH (categorical variables) and NAFLD was assessed using a binary logistic regression model, and results were expressed as odds ratios (OR) with a 95% confidence interval (CI). To evaluate the potential for confounding, a series of models were built. In model 1, we adjusted for age. Model 2 included the variables in model 1, plus BMI and WC. Model 3 included the variables in model 2 plus education level, marital status, smoking habits, routine exercise, history of hypertension and diabetes, and medication usage (anti-hypertension or lipid-lowering). Model 4 further adjusted for the scores of MNA-SF in addition to the variables in model 3. Model 5 further adjusted for the quartiles of E_2_, TT, and LH in addition to the variables in model 4. For assessing the association between FSH and FIB-4 index, the Pearson correlation analysis was conducted in data from patients with NAFLD. A multi-adjusted binary logistic regression model was used to investigate the independent relationship between FSH and advanced fibrosis (defined as FIB-4 index > 2.67) after adjusting for potential covariates associated with hepatic fibrosis. All statistical analyses were performed using SPSS 26.0 for Mac (IBM, Chicago, IL). A two-sided *P*-value < 0.05 was considered statistically significant.

## Results

### Characteristics of the study population

The clinical characteristics of the study participants are presented in Table 1. This study recruited 444 men of minimal 80 years of age. Among them, 108 (24.3%) participants were diagnosed with NAFLD based on their ultrasound.

**Table 1:** Characteristics of all 444 subjects according to NAFLD

	Non-NAFLD	NAFLD	*P*
N	336	108	
Age (years)	86 (83–90)	84 (82–88)	0.006
BMI (kg/m^2^)	23.6 ± 3.3	26.0 ± 3.0	< 0.001
WC (cm)	89 ± 9	97 ± 8	< 0.001
SBP (mmHg)	134 ± 21	136 ± 19	0.202
DBP (mmHg)	69 ± 12	69 ± 12	0.986
Diabetes (%)	36.9	41.5	0.394
Hypertension (%)	79.8	94.4	< 0.001
Current/ex-smoker (%)	27.6	26.5	0.827
High education (%)	80.0	86.5	0.135
Married status (%)	92.7	90.6	0.481
Routine exercise^a^ (%)	41.3	44.2	0.599
MNA-SF score	12.0 (10.0–13.3)	13.0 (12.0–14.0)	< 0.001
Malnutrition/Nutritional Risk (%)	36.3	13.2	< 0.001
Lipid-lowering drug users (%)	51.2	64.8	0.013
Antihypertensive drug users (%)	61.3	75.9	0.006
TT (nmol/L)	12.9 (8.5–18.4)	9.5 (6.5–11.7)	< 0.001
E_2_ (pmol/L)	140 (106–167)	113 (83–136)	< 0.001
LH (IU/L)	10.1 (6.8–21)	9.5 (6.5–11.7)	0.568
FSH (IU/L)	17.8 (12.7–35.2)	22.4 (14.2–42.9)	0.025
HbA1c (%)	5.7 (5.5–6.2)	6.2 (5.8–6.8)	< 0.001
FPG (mmol/L)	5.0 (4.7–5.6)	5.6 (5.2–6.5)	< 0.001
FINS (mU/L)	5.9 (4.0–9.4)	11.0 (8.1–14.6)	< 0.001
TC (mmol/L)	3.84 (3.22–4.64)	4.01 (3.52–4.71)	0.086
TG (mmol/L)	0.90 (0.65–1.25)	1.34 (1.05–2.08)	< 0.001
LDL-c (mmol/L)	2.03 (1.64–2.72)	2.33 (1.86–2.79)	0.002
HDL-c (mmol/L)	1.15 (0.94–1.40)	0.96 (0.86–1.19)	< 0.001
ALT (U/L)	12 (9–18)	15 (11–20)	0.004
AST (U/L)	18 (15–22)	19 (17–22)	0.082
hsCRP (mg/L)	1.34 (0.49–4.56)	1.47 (0.81–2.72)	0.555
PCT (ng/mL)	0.036 (0.026–0.047)	0.041 (0.029–0.054)	0.007

Data were summarized as the mean ± standard deviation for normally distributed variables or median (interquartile range) for skewed variables continuous variables or as proportion for categorical variables. For normally distributed variables and skewed variables, the differences were evaluated with the Student’s t-test or Mann–Whitney U-test. The chi-square (χ^2^) test was used for categorical variables. ^a^Routine exercise: moderate to strenuous intensity ≥3 times a week

*Abbreviations*: *ALT* Alanine aminotransferase, *AST* Aspartate aminotransferase, *BMI* body mass index, *DBP* diastolic blood pressure, *E*_*2*_ estradiol, *FINS* fasting insulin, *FPG* fasting plasma glucose, *FSH* follicle-stimulating hormone, *HbA1c* hemoglobin A1c, *HDL-c* high-density lipoprotein cholesterol, *hsCRP* high sensitive C-reactive protein, *LDL-c* low-density lipoprotein cholesterol, *LH* luteinizing hormone, *MNA-SF* Short-form of Mini Nutritional Assessment, *PCT* procalcitonin, *SBP* systolic blood pressure, *TC* total cholesterol, *TG* triglyceride, *TT* total testosterone, *WC* waist circumference

The two groups stratified by NAFLD status did not differ with respect to systolic blood pressure (SBP), diastolic blood pressure (DBP), total cholesterol (TC), AST, and hsCRP. There was also no difference in the frequency of diabetes, ex-smokers, high education, married status, and routine exercise between the two groups. Elderly subjects with NAFLD were younger and had worse indicators of glycolipid metabolism, reflected as significantly lower high-density lipoprotein cholesterol (HDL-c) and higher BMI, WC, fasting plasma glucose (FPG), FINS, HbA1c, triglyceride (TG), and low-density lipoprotein cholesterol (LDL-c), than those without NAFLD. Unexpectedly, these subjects also had higher levels of the liver enzyme ALT, an index of liver injury induced by NAFLD (all *P* < 0.01). They also had lower nutritional risk manifested as higher scores of the MNA-SF and the proportion of malnutrition (*P* < 0.01, Table 1). As for sex hormones, NAFLD subjects had higher levels of FSH [22.4 (14.2–42.9) vs. 17.8 (12.7–35.2) IU/L, *P* < 0.05] but lower levels of TT and E_2_ than those without the condition (both *P* < 0.01), with no significant differences in circulating LH levels (Table 1).

The clinical characteristics of participants subdivided by FSH quartiles are shown in Table 2. The quartile ranges of FSH were ≤ 12.96 (Q1), 12.97–18.99 (Q2), 19.00–38.59 (Q3), ≥38.60 IU/L (Q4). Compared with subjects in the lowest quartile (Q1), the population in the highest quartile (Q4) had comparable BMI, WC, SBP, DBP, HbA1c, FPG, FINS, TC, LDL-c, ALT, AST, hsCRP, PCT, and nutritional risk, but a higher age (*P* < 0.01), TG (*P* < 0.05), and a lower education level (*P* < 0.01). These subjects had higher LH (*P* < 0.01) but significantly lower E_2_ and TT levels (both *P* < 0.01). No statistically significant differences were observed between populations in the Q1 and Q4 groups in the proportion of diabetes, hypertension, ex-smokers, married status, routine exercise, and medication usage (lipid-lowering and antihypertensive drugs).

**Table 2:** Characteristics of the four subject groups, stratified by FSH

Variable	Q1	Q2	Q3	Q4	*P*
N	110	110	110	114	
FSH (IU/L)	9.61 (7.40–11.41)	14.82 (13.96–16.94)	25.12 (21.43–32.35)	51.6 (42.85–64.94)	
Age (years)	84 (81–87)	85 (83–89)	85 (83–89)	88 (85–91)	< 0.001
BMI (kg/m^2^)	24.3 ± 3.0	25.0 ± 3.5	23.5 ± 3.4	24.0 ± 3.6	0.020
WC (cm)	91 ± 8	93 ± 8	90 ± 11	90 ± 11	0.014
SBP (mmHg)	137 ± 22	133 ± 22	133 ± 19	134 ± 19	0.425
DBP (mmHg)	71 ± 12	70 ± 12	66 ± 11	70 ± 11	0.030
Diabetes (%)	34.5	36.4	47.3	35.1	0.166
Hypertension (%)	85.5	78.2	89.1	80.7	0.130
Current/ex-smoker (%)	24.5	25.5	25.9	33.3	0.426
High education (%)	87.8	88.9	88.5	63.2	< 0.001
Married status (%)	92.2	94.4	92.7	89.5	0.579
Routine exercise^a^	44.9	40.0	41.2	42.1	0.912
MNA-SF score	13.0 (11.0–14.0)	13.0 (12.0–14.0)	12.0 (11.0–13.0)	12.0 (10.0–14.0)	0.034
Lipid-lowering drug users (%)	50.9	52.7	58.2	56.1	0.697
Antihypertensive drug users (%)	60	60	70.9	68.4	0.197
TT (nmol/L)	14.7 (8.0–19.5)	13.3 (9.3–18.4)	10.4 (7.9–15.0)	9.3 (6.3–12.6)	< 0.001
E_2_ (pmol/L)	141 (124–174)	153 (119–188)	121 (99–152)	109 (89–143)	< 0.001
LH (IU/L)	6.0 (4.7–8.3)	8.5 (6.2–10.5)	12.1 (8.1–17.3)	25.7 (22.3–35.8)	< 0.001
HbA1c (%)	5.7 (5.6–6.1)	5.9 (5.4–6.7)	6.0 (5.6–6.5)	5.9 (5.5–6.2)	0.191
FPG (mmol/L)	5.2 (4.6–5.7)	5.1 (4.8–5.8)	5.4 (4.8–6.4)	5.2 (4.8–5.6)	0.373
FINS (pmol/L)	6.4 (4.3–10.5)	8.1 (5.2–11.1)	7.1 (4.0–12.0)	6.8 (4.8–11.3)	0.312
TC (mmol/L)	3.74 (3.22–4.46)	4.06 (3.30–4.70)	4.00 (3.34–4.94)	3.89 (3.39–4.39)	0.143
TG (mmol/L)	0.83 (0.61–1.19)	1.08 (0.81–1.45)	1.04 (0.68–1.36)	1.07 (0.83–1.42)	0.004
LDL-c (mmol/L)	2.00 (1.70–2.47)	2.24 (1.63–2.89)	2.07 (1.71–2.97)	2.03 (1.64–2.60)	0.110
HDL-c (mmol/L)	1.14 (0.92–1.44)	1.10 (0.90–1.22)	1.04 (0.88–1.35)	1.20 (0.97–1.36)	0.115
ALT (U/L)	12 (10–18)	14 (10–20)	13 (8–19)	12 (8–19)	0.389
AST (U/L)	18 (16–22)	20 (17–23)	18 (15–22)	20 (15–23)	0.207
hsCRP (mg/L)	1.35 (0.46–5.58)	1.23 (0.58–2.04)	1.47 (0.58–3.34)	1.40 (0.49–3.64)	0.583
PCT (ng/mL)	0.037 (0.024–0.042)	0.037 (0.026–0.045)	0.036 (0.023–0.50)	0.039 (0.030–0.054)	0.088

Data were summarized as the mean ± standard deviation for normally distributed variables or median (interquartile range) for skewed variables continuous variables or as proportion for categorical variables. The Kruskal–Wallis test and ANOVA were used for continuous variables with a skewed or normal distribution, and the chi-square (χ^2^) test was used for categorical variables. ^a^Routine exercise: moderate to strenuous intensity ≥3 times a week

*Abbreviations*: *ALT* Alanine aminotransferase, *AST* Aspartate aminotransferase, *BMI* body mass index, *DBP* diastolic blood pressure, *E*_*2*_ estradiol, *FINS* fasting insulin, *FPG* fasting plasma glucose, *FSH* follicle-stimulating hormone, *HbA1c* hemoglobin A1c, *HDL-c* high-density lipoprotein cholesterol, *hsCRP* high sensitive C-reactive protein, *LDL-c* low-density lipoprotein cholesterol, *LH* luteinizing hormone, *MNA-SF* Short-form of Mini Nutritional Assessment, *PCT* procalcitonin, *SBP* systolic blood pressure, *TC* total cholesterol, *TG* triglyceride, *TT* total testosterone, *WC* waist circumference

### Correlation of FSH with covariates that were different between FSH quartile groups

As shown in Table 3, FSH level were negatively correlated with the MNA-SF score, TT, and E_2_ (*r* = − 0.143, − 0.276, and − 0.303, respectively, all *P* < 0.01), the prevalence of high education (ρ = − 0.221, *P* < 0.01), and positively correlated with age, LH, ALT, and AST (*r* = 0.252, 0.800, 0.119, and 0.156, respectively, all *P* < 0.05). However, FSH was not significantly correlated with WC, DBP, or TG. The correlation between FSH and BMI or antihypertensive drug usage was marginally significant (*r* = − 0.092, *P* = 0.057; ρ = 0.087, *P* = 0.066, respectively).

**Table 3:** Pearson and Spearman correlation of FSH with variates which were different among FSH quartile group

	*r*/*ρ*	*P*
Age	0.252	< 0.001
BMI	−0.092	0.057
WC	−0.081	0.101
DBP	0.024	0.608
High education	−0.221	< 0.001
Antihypertensive drug usage	0.087	0.066
MNA-SF score	−0.143	0.003
TT	−0.276	< 0.001
E_2_	−0.303	< 0.001
LH	0.800	< 0.001
TG	0.048	0.320
ALT	0.119	0.012
AST	0.156	0.001

Pearson or spearman analysis was used to assess the relationship between FSH level and the various parameters

*Abbreviations*: *ALT* Alanine aminotransferase, *AST* Aspartate aminotransferase, *BMI* body mass index, *DBP* diastolic blood pressure, *E*_*2*_ estradiol, *IADL* instrumental activities of daily living scales, *LH* luteinizing hormone, *MNA-SF* Short-form of Mini Nutritional Assessment, *TG* triglyceride, *TT* total testosterone, *WC* waist circumference

### Prevalence of NAFLD

**Fig. 2:**
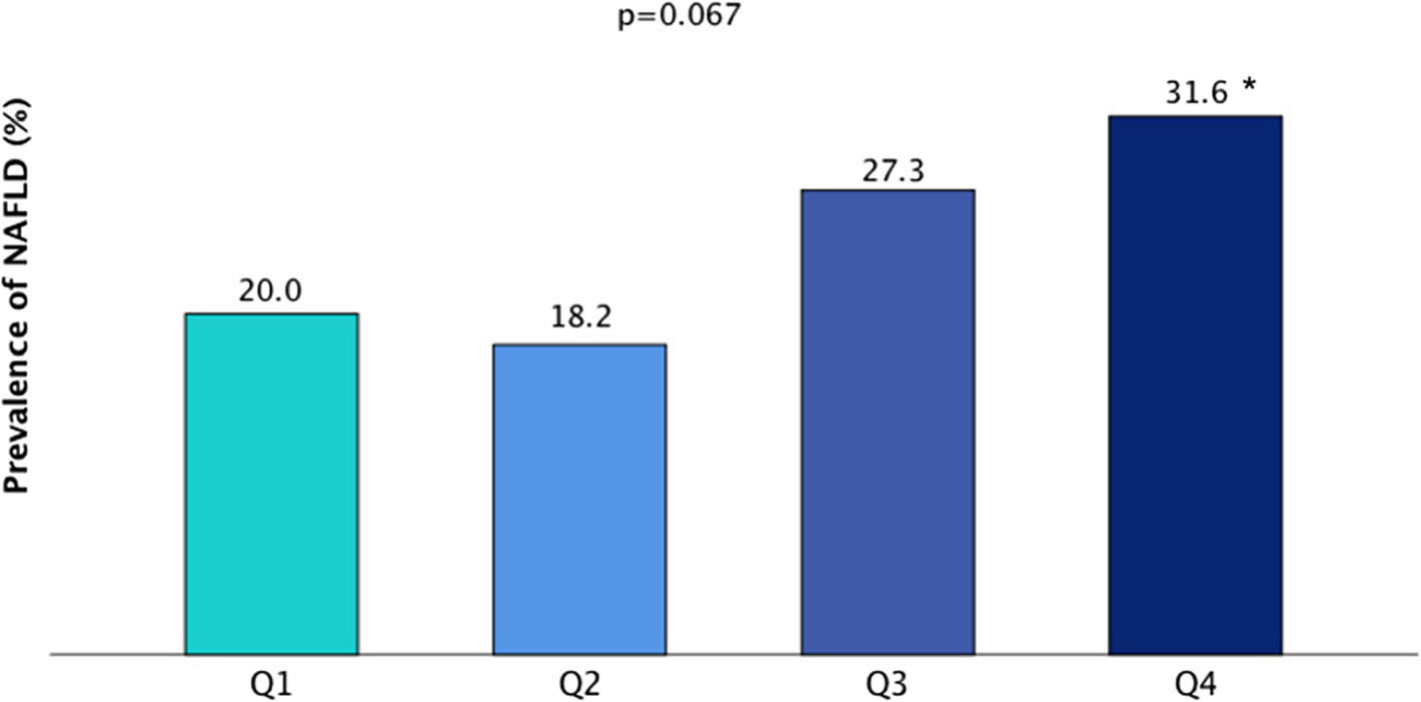
Prevalence of NAFLD according to serum FSH quartile categories. Notes: *P* value was analyzed using the chi-square (χ^2^) test. Range of quartile 1 (Q1), ≤ 12.96 IU/L; quartile 2 (Q2), 12.97–18.99 IU/L; quartile 3 (Q3), 19.00–38.59 IU/L; quartile 4 (Q4), ≥ 38.60 IU/L. *: Q1 vs Q4, *P* = 0.048

Figure 2 demonstrates the proportion of NAFLD cases in each of the serum FSH quartiles. The percentage of subjects with NAFLD gradually increased following the quartiles of serum FSH. 20.0% in Q1, 18.2% in Q2, 27.3% in Q3, and 31.6% in Q4 had NAFLD based on ultrasound results. The differences between the proportion of NAFLD among FSH quartiles was not significant (*P* = 0.067), but the prevalence of NAFLD in Q4 was significantly higher than that in Q1 (*P* = 0.048).

### Association between FSH levels and NAFLD

As we found an increased trend in NAFLD proportions among FSH quartiles, we investigated the potential association of FSH with NAFLD by constructing a binary logistic regression model. As shown in Table 4, there were significant associations between the serum FSH quartiles and NAFLD in our very elderly population. The OR of NAFLD in Q1 relative to that in Q4 was 0.405 (95% CI: 0.212–0.773) in model 1 adjusted for age. Since blocking FSH was reported to reduce body fat in mice [18], we next determined whether obesity was involved in the potential negative role of circulating FSH on the risk of NAFLD by further adjusting for BMI and WC in model 2. The result did not change (OR: 0.363, 95% CI: 0.175–0.753). Similar results were obtained after additional adjustments for demographic confounders, lifestyle, history of hypertension and diabetes, and medicine usage (model 3; OR: 0. 336, 95% CI: 0.158–0.714), or covariates in model 3 plus MNA-SF (model 4; OR: 0.303, 95% CI: 0.141–0.653). We did not observe a significant dose-dependent response between serum FSH quartiles and NAFLD in model [1–4]. Accumulating evidence suggests that sex steroid hormones are critical regulators in individuals with a predisposition for NAFLD [9, 14, 19]. In our fully adjusted model (model 5), other sex hormones E_2_, TT, and LH were also included, and to our surprise, in this model serum FSH were progressively associated with ORs for NAFLD. The adjusted ORs and 95% CIs for Q1, Q2, and Q3, compared with Q4 were 0.132 (0.034–0.516), 0.190 (0.052–0.702), and 0.404 (0.139–1.173), respectively.

**Table 4:** The association between serum FSH level and NAFLD in this study

	Odds ratios	95% confidence intervals	*P* value
Model 1			
Q1	0.405	0.212–0.773	0.006
Q2	0.400	0.210–0.762	0.005
Q3	0.665	0.366–1.209	0.181
Q4	1	/	/
Model 2			
Q1	0.363	0.175–0.753	0.006
Q2	0.316	0.151–0.662	0.002
Q3	0.713	0.366–1.388	0.320
Q4	1	/	/
Model 3			
Q1	0.336	0.158–0.714	0.005
Q2	0.274	0.124–0.602	0.001
Q3	0.519	0.255–1.053	0.069
Q4	1	/	/
Model 4			
Q1	0.303	0.141–0.653	0.002
Q2	0.260	0.118–0.573	0.001
Q3	0.498	0.241–1.029	0.060
Q4	1	/	/
Model 5			
Q1	0.132	0.034–0.516	0.003
Q2	0.190	0.052–0.702	0.011
Q3	0.404	0.139–1.173	0.075
Q4	1	/	/

Model 1: Adjusted for age

Model 2: Adjusted for age, BMI, and WC

Model 3: Adjusted for the covariates in model 2 plus education level, marital status, smoking habit, routine exercise, history of hypertension and diabetes, medication usage (antihypertension or lipid-lowering)

Model 4: Adjusted for the covariates in model 3 plus score of the MNA-SF

Model 5: Adjusted for the covariates in model 4 plus quartiles of E_2_, TT, and LH

**Fig. 3:**
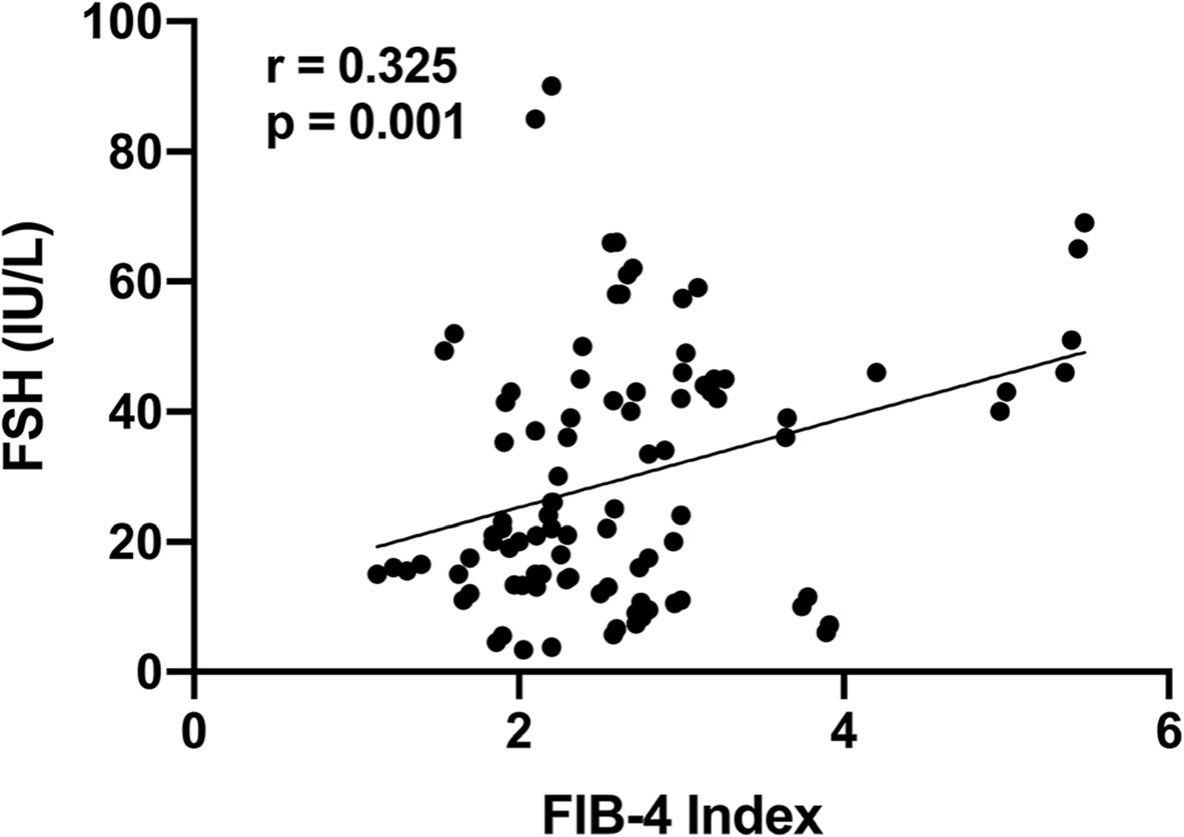
Pearson correlation analysis of FSH and FIB-4 index in subjects with NAFLD. Notes: FIB-4 (Fibrosis-4) index was calculated by the formula: age (yrs) × AST[U/L] / (platelet[10^9^/L] × (ALT[U/L]^1/2^)

The presence of advanced fibrosis in NAFLD is mostly associated with adverse outcomes that make advanced fibrosis clinical paramount in disease management. Therefore, we further investigated the association between FSH and advanced fibrosis in subjects with NAFLD. We first conducted the Pearson correlation analysis between FSH and FIB-4 index, an accurate and non-invasive marker for identifying/excluding advanced fibrosis. As shown in Fig. 3, FSH positively correlated with FIB-4 index (*r* = 0.325, *P* = 0.001). Then, a multi-adjusted logistic regression model was used to investigate the independent relationship between FSH and advanced fibrosis (defined as FIB-4 > 2.67). The results revealed no significant association between FSH and advanced fibrosis, and the OR (95% CI) for advanced fibrosis was 1.018 (0.983–1.054) (*P* = 0.316) after adjusting for the covariates associated with hepatic fibrosis, such as age, BMI, WC, smoking habit, FINS, history of diabetes, TG and E_2_.

## Discussion

As previously reported, a consensus by 32 experts from 22 different countries has proposed to rename NAFLD as metabolic associated fatty liver disease (MAFLD) with positive diagnostic criteria rather than an exclusion diagnosis recently [20, 21]. This single letter change provides a better understanding of the disease from patients’ point of view due to the inclusion of “metabolic” in it. Furthermore, as a reproductive hormone associated with metabolic disorders in human beings, FSH was observed to associate with an increased risk of NAFLD in older men for the first time. And, this association was independent of age, demographic factors, lifestyle factors, history of hypertension and diabetes, BMI, WC, nutritional risk, and other sex hormones. However, no significant association between FSH and advanced fibrosis (defined as FIB-4 > 2.67) was found in the present study.

Prior data investigating FSH in metabolic diseases, including NAFLD, have primarily focused on postmenopausal women [10–14]. Indeed, FSH is more often measured and concerned in females, and postmenopausal women have higher metabolic risks than pre-menopausal women. Our study represents an important advance in our understanding of FSH and NAFLD, highlighting the association of FSH levels in an aging male population with the risk of NAFLD.

Few other studies have looked at FSH levels in relation to NAFLD in males or postmenopausal women. In line with our findings, a study of community-dwelling men aged 20–69 years observed a progressive increase in FSH with aging [9]. However, no association between FSH and NAFLD was found, which may be explained by the limited adjustment for only age, LDL-c, and other sex hormones in the logistic regression analyses. In contrast with our data, a previous study conducted in a postmenopausal female population revealed that FSH was anti-steatotic, with low FSH conferring a higher risk of ultrasonography-confirmed NAFLD [14]. It remains unknown why FSH plays sex-dependent roles predisposing individuals to NAFLD, although androgens may also affect men and women differently [7, 22].

The precise mechanism behind the association of FSH and NAFLD remains unknown. FSH is now regarded to play direct roles in a variety of nongonadal tissues by interacting with the FSH receptors in those tissues [23–25]. For example, FSH promotes fat accumulation and fat redistribution (from subcutaneous fat depots to visceral fat depots) in aging, and blocking FSH with polyclonal antibodies induces thermogenic adipose tissue and reduces body fat [18, 26]. The positive association between FSH and obesity was confirmed in human studies in which circulating FSH levels were observed to correlate with increases in BMI of both males and females [26]. Notably, we failed to find this association in our elderly male population, possibly because non-obese NAFLD (BMI < 28 kg/m^2^) was more common (81.1%) in this population and BMI is a poor predictor of adiposity in some elders with height loss. Our findings from epidemiological investigations seem suggest that FSH also drive ectopic (such as liver) fat deposition besides its regulation in adipose tissue metabolism, although no evidence of direct association between FSH and hepatis steatosis was reported previously. Considering the aging-associated changes in circulating FSH level and prevalence of NAFLD, elevated FSH may be one of the possible mechanisms to explain the more NAFLD subjects found in elderly. Further large prospective studies and basic research are required to confirm our hypothesis in the future.

We did not find any association between FSH and advanced fibrosis (defined as FIB-4 > 2.67), although a positive correlation of FSH and FIB-4 index was observed. It is well known that non-invasive scores are being increasingly used to screen for advanced fibrosis in NAFLD and FIB-4 score classify advanced fibrosis dichotomously with two cut-offs. A cut-off of < 1.3 indicates low risk for advanced fibrosis, and > 2.67 indicates a high risk of advanced fibrosis. The rest of the patients are classified as having an indeterminate risk for advanced fibrosis [27]. Whilst, it has been proposed recently that its leading clinical utility in patients with NAFLD lies in the ability to exclude, not identify, advanced fibrosis [28–30]. Furthermore, age has been proposed as a confounding factor for the accurate non-invasive diagnosis of advanced NAFLD fibrosis [31]. Whether the proposed cut-off for high risk of advanced fibrosis (FIB-4 > 2.67) also perform well in older men aged over 80 remained to be investigated in the future.

Dietary protein deficiency has been associated with excessive TG storage and NAFLD in population studies, and older adults are at risk for protein malnutrition [32]. Therefore, in addition to common pathogenic risks of NAFLD, we also included nutritional risk as a variable in our analysis of the association between FSH and NAFLD. Using the scores of the MNA-SF, we showed that participants in the present study had a high prevalence of malnutrition/nutritional risk (30.5%). However, to our surprise, elderly subjects with NAFLD had a lower nutritional risk than those who were non-NAFLD (Table 1). This lack of association between protein insufficiency and NAFLD may be due to adequate protein intake in the studied population who lived in Shanghai, a large city with a thriving economy. Nevertheless, a possible negative correlation of circulating FSH and cores of MNA-SF was observed in this study (after adjusting for age, *r* = − 0.092, *P* = 0.059), and cores of MNS-SF were included in the multivariate logistic regression analysis.

Limitations of this study include that the subjects are from a population receiving regular medical examination as opposed to a community or a general population, so our results may not reflect the general population. Liver ultrasonography but not a liver biopsy was used to diagnose NAFLD in our subjects. This was chosen since healthy subjects would likely not accept to perform liver biopsies routinely. Classification of the severity of ultrasound features was not available in the present work due to the study design limitations and thus led to the impossibility for analyzing the effect of FSH on the severity of NAFLD. Instead, we used the FIB-4 index, a simple and non-invasive marker for identifying advanced fibrosis. This was a cross-sectional single-center study with a relatively small sample size, which is a significant flaw, and further prospective studies with larger sample sizes are required to confirm our present findings.

## Conclusion

In conclusion, FSH levels were positively associated with the risk of NAFLD in old males aged over 80. Common pathogenic risks of NAFLD, nutritional risk and other sex hormones, could not explain this association between NAFLD and FSH.

## Data Availability

Not applicable.

## Abbreviations

ALT: Alanine aminotransferase
ANOVA: One-way analysis of the variance
AST: Aspartate aminotransferase
BMI: Body mass index
CI: Confidence interval
DBP: Diastolic blood pressure
E_2_: Estradiol
FIB-4: Fibrosis-4
FINS: Fasting serum insulin
FPG: Fasting plasma glucose
FSH: Follicle-stimulating hormone
HbA1c: Hemoglobin A1c
HDL-c: Lower high-density lipoprotein cholesterol
hsCRP: High-sensitivity C-reactive protein
LDL-c: Low-density lipoprotein cholesterol
LH: Luteinizing hormone
MNA-SF: The Short-form of Mini Nutritional Assessment
NAFLD: Non-alcoholic fatty liver disease
OR: Odds ratios
PCT: Procalcitonin
SBP: Systolic blood pressure
SD: Standard deviation
TC: Total cholesterol
TG: Triglyceride
TT: Total testosterone
WC: Waist circumference
